# Male Circumcision Revisited: A Narrative Review of Techniques, Therapeutic Indications, and Preventive Benefits

**DOI:** 10.7759/cureus.98581

**Published:** 2025-12-06

**Authors:** Muhammad Rakib Hasan, Asmita Hossain, Nazmus Sakib

**Affiliations:** 1 Urology, Watford General Hospital, West Hertfordshire Hospitals NHS Foundation Trust, Watford, GBR; 2 Urology, Surrey and Sussex Healthcare NHS Trust, Redhill, GBR; 3 Gastroenterology and Hepatology, Square Hospitals Limited, Dhaka, BGD

**Keywords:** balanitis, circumcision techniques, hiv prevention, human papillomavirus, male circumcision, pediatric urology, penile cancer, phimosis, urinary tract infection

## Abstract

Male circumcision (MC) is a common pediatric operation and is undertaken for religious, cultural, therapeutic, and preventive reasons. Over the past two decades, MC has been investigated as a method that may reduce heterosexual acquisition of HIV and other sexually transmitted infections, lower the risk of penile carcinoma, and decrease urinary tract infections in early childhood. This narrative review revisits MC by integrating embryologic and anatomic concepts with contemporary operative techniques, therapeutic indications, and proven preventive benefits across age groups. Conventional open procedures (including dorsal slit, sleeve resection, and forceps-guided circumcision) and device-based methods (such as Plastibell {Caledonia, MI: Aspen Surgical Products, Inc.} and other ring devices) are summarized with emphasis on appropriate anesthesia, perioperative care, and prevention and management of complications. MC is an effective definitive treatment for pathologic phimosis, recurrent balanitis, selected cases of balanitis xerotica obliterans, and recurrent urinary infections, and it may be considered in specific high-risk urologic and dermatologic conditions. Beyond these therapeutic indications, robust data support MC as a durable preventive intervention. When performed by trained providers using standardized techniques, adequate analgesia, and sterile conditions, MC is a safe procedure with predominantly minor, early complications and very low rates of severe adverse events. Ongoing research is needed to refine device selection, expand access in low-resource settings, and clarify long-term functional and sexual outcomes to inform shared decision-making among patients, families, clinicians, and public health policymakers.

## Introduction and background

The estimated worldwide rate of male circumcision is approximately 36.7-38.7% [1,2]. A significant proportion of circumcisions are carried out in response to deeply ingrained religious or cultural mandates. Specifically, certain communities, notably the Jewish and Muslim populations, demonstrate a circumcision prevalence that is close to 100% [1]. Among sub-Saharan African nations, the highest rates of male circumcision are predominantly observed in countries with a Muslim majority [2]. In or around the year 2010, the provision of voluntary medical male circumcision (VMMC) was introduced in additional countries. This initiative was implemented as a key component within a broader range of strategies aimed at preventing the transmission of HIV. The rollout of these programs was significantly supported by multilateral organizations, including the World Health Organization (WHO), the Joint United Nations Programme on HIV/AIDS (UNAIDS), and the United States President’s Emergency Plan for AIDS Relief (PEPFAR). This collective support was instrumental in driving the expansion of VMMC as a public health intervention [3].

Strong evidence now exists demonstrating that MC lowers a man's risk of contracting HIV. According to predictive models, scaling up MC programs in high-prevalence African nations could prevent as many as two million deaths over the next decade, prompting several of these countries to expand their public health MC initiatives [4]. Circumcision is the main treatment for certain pathological conditions like phimosis, although preputioplasty offers a viable alternative in carefully chosen instances [5,6]. Reported methods of circumcision are diverse, offering unique advantages and associated risks of complications [4,7]. The incidence of complications is influenced by several factors, such as anatomical issues, existing medical conditions, the surgical method used, and the patient's age [8]. When circumcision is performed in clinical settings to treat phimosis, the dorsal slit technique is frequently chosen because the main objective is to uncover the glans penis [9,10].

## Review

The development of the penis begins around the seventh week of gestation and is finished by the 17th week. The skin at the front of the penis folds over to create the prepuce, or foreskin, which covers the glans penis and the urinary opening (meatus). This double-layered fold, which spans about 15 square inches, has several functions, primarily protection, immune defense, and erogenous sensation. The inner mucosal layer of the foreskin joins with the glans [11,12]. 

The underside of the glans is attached to the prepuce by a very sensitive tissue called the frenulum. The prepuce has many blood vessels and nerves, with abundant fine touch receptors. Standard circumcision typically removes most of these sensitive areas [13]. The glans penis contains only pressure receptors, lacking the fine touch receptors found in the prepuce (foreskin). Both the prepuce and glans secrete substances that help with lubrication and offer protection against infection. These secretions contain lysozyme, which fights harmful microbes, as well as Cathepsin B, chymotrypsin, neutrophil elastase, cytokines, and pheromones like androsterone. The presence of Langerhans cells in the prepuce appears to contribute to resistance against HIV infection [12,14].

Techniques for circumcision

Circumcision is typically carried out using general or local anesthesia, although sometimes it is done without any anesthesia, particularly for religious or cultural reasons. Local anesthesia can be administered through methods like a penile ring block or a penile dorsal nerve block [15].

Shield and Clamp

Using this approach, the foreskin is drawn forward beyond the glans, and a protective metal shield is placed over the foreskin, right next to the glans. The excess foreskin beyond the shield is then removed with a scalpel. This shield safeguards the glans, and the frenulum is left intact during the removal process. The inner layer of the foreskin can then be incised and removed, extending behind the glans to guarantee complete glans exposure after the healing process. No sutures are required; the wound is merely bandaged for bleeding control (hemostasis). The glans and the frenulum are protected from the surgical instrument, preventing damage. While injury to the glans or a urethrocutaneous fistula is rare, significant bleeding is a notable risk with this technique [15].

Plastibell

The technique involves inserting a plastic bell with a groove between the glans and the foreskin, often requiring an initial dorsal slit for placement. The foreskin is then pulled forward slightly, and a suture is tightly tied around it within the bell's groove. This tight ligation stops the blood flow to the distal foreskin, causing it to necrose and fall off within seven to 10 days [16].

Gomco

The procedure involves retracting the foreskin fully, placing a metal bell over the glans, and then bringing the foreskin back over the bell (often facilitated by a dorsal slit). A metal plate, designed to fit around the bell's rim, is placed over the bell, trapping the foreskin between the bell and the plate. A tensioning bar is then used to screw the plate tightly onto the bell, securing the foreskin. The foreskin is then excised by running a scalpel around the plate's upper edge after sufficient strangulation has been achieved. Bleeding is a potential complication, necessitating hemostatic stitches. Using diathermy during this procedure carries the risk of severe damage, potentially leading to the loss of the entire penis. A key advantage, shared with other shield methods, is the protection offered to both the glans and the frenulum [17-20].

PrePex

The PrePex device (Herzliya, Israel: Circ MedTech) is a device-based method for adult male circumcision that does not require injected local anesthesia for application. It consists of a placement ring, an inner ring, and an elastic ring. When in place, the foreskin is compressed between the inner ring and elastic ring, resulting in ischemic necrosis of the distal foreskin; the device is typically removed about one week later and the nonviable foreskin is excised. Because elastic-collar compression device methods leave nonviable tissue in situ for several days, WHO has issued updated safety guidance (including tetanus prevention requirements); therefore, any use of PrePex should follow current WHO and national guidance and appropriate tetanus immunization practices [21,22].

Forceps-Guided Circumcision

This technique involves pulling the foreskin forward, away from the glans. A strong, locking pair of forceps is then clamped across the foreskin, parallel to the coronal margin and positioned just distal to the glans. The foreskin is removed by cutting along the line of the forceps with a scalpel, with the instrument acting as a shield to protect the glans. The method is conceptually similar to the guillotine technique. WHO guidance for voluntary medical male circumcision describes forceps-guided circumcision as one of the three standard surgical methods) and emphasizes performance by trained providers under local anesthesia with sterile technique and careful hemostasis [15,23,24].

Dorsal Slit Circumcision

**Figure 1:**
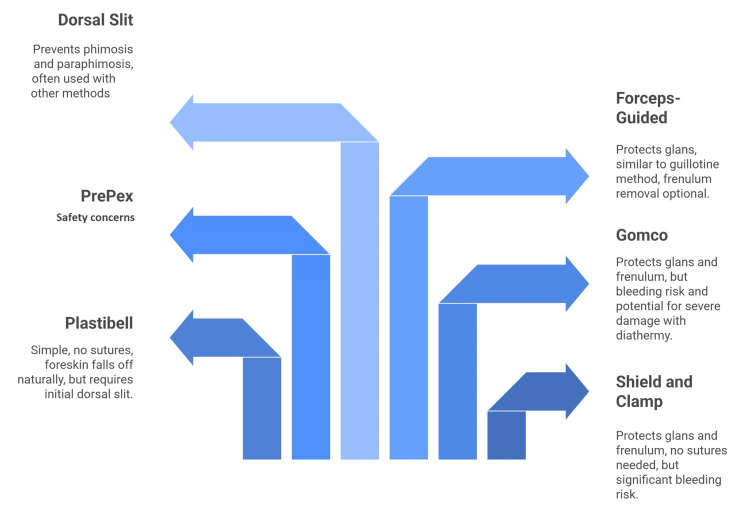
Techniques for circumcision. The image is created by the authors of this study.

The dorsal slit is a procedure frequently incorporated into various circumcision techniques, and sometimes used on its own, particularly when acute inflammation is present. Its purpose is to prevent both phimosis and paraphimosis. The method involves first separating the foreskin from the glans due to adhesions. Then, using artery forceps positioned at 10 and 1 o'clock, a cut is made at the 12 o'clock position through both layers of the foreskin, extending a few millimeters past the corona. While many other circumcision methods begin with a dorsal slit to enlarge the outer preputial opening, performing the dorsal slit alone, without excising the foreskin, is usually considered to have an unsatisfactory cosmetic result (Figure 1) [15-26].

Therapeutic indications

Phimosis is the most common therapeutic indication for circumcision, accounting for approximately 46.5% of adult circumcisions. Phimosis is characterized by an inability to retract the foreskin due to a narrow preputial ring, resulting in urinary difficulties and pain during sexual activity. While topical corticosteroid therapy achieves success rates between 62% and 87%, circumcision remains the definitive surgical treatment when conservative management fails. Current clinical practice recommends attempting medical management with potent topical steroids combined with manual stretching for six to eight weeks before resorting to surgery [27,28].

Paraphimosis represents a urological emergency where the retracted foreskin becomes entrapped behind the glans, causing constriction and swelling. While initial management involves manual reduction with sedation and anesthesia, circumcision is the definitive therapy to prevent recurrent episodes. The dorsal slit procedure may be performed acutely to relieve constriction, but definitive circumcision should be performed subsequently to prevent recurrence [29,30].

Balanitis and balanoposthitis (inflammation of the glans and foreskin, respectively) represent common indications for circumcision, particularly when recurrent or resistant to medical management. These conditions account for approximately 14.4% of adult circumcisions, with recurrent balanoposthitis being a relative indication when non-surgical interventions, such as topical antifungals and corticosteroids, prove ineffective [27,31].

Reducing risks of sexually transmitted infections

Male circumcision is a procedure characterized by its low cost, minimal invasiveness, and high degree of cultural acceptance in numerous societies. Importantly, this procedure has been scientifically proven to be an effective intervention for significantly decreasing the risk of sexual transmission of the human immunodeficiency virus (HIV). Furthermore, it has also demonstrated efficacy in lowering the rates of other common sexually transmitted infections (STIs) [32].

Across a substantial body of research, including rigorous systematic reviews and comprehensive meta-analyses, both randomized controlled trials (RCTs) and various observational studies have reached a consistent conclusion, i.e., male circumcision significantly lowers the likelihood of acquiring the human immunodeficiency virus (HIV). Specifically, this body of evidence demonstrates that male circumcision provides a protective effect, reducing the risk of HIV infection by an estimated range of 40-60% [33-35]. As a direct result of this evidence, the World Health Organization (WHO) and the Joint United Nations Programme on HIV/AIDS (UNAIDS) have officially recommended voluntary medical male circumcision (VMMC) since 2007. This procedure is now considered an integral component of a comprehensive set of strategies aimed at preventing HIV transmission. Crucially, VMMC is intended to be implemented alongside, and to complement, a range of other established HIV prevention methods, which include various behavioral changes, structural interventions, and pharmacological (drug-based) approaches [3,35].

Understanding the biological way in which male circumcision (MC) lowers the chance of contracting HIV is crucial. This understanding not only strengthens the reliability of data from epidemiological studies but could also reveal new possibilities for preventative strategies. HIV infects cells by attaching to receptors found on Langerhans cells [36-39]. While most HIV is eliminated within Langerhans cells, each exposure carries a slight risk that the viral load will overcome these killing mechanisms. If this happens, the Langerhans cells proceed to their role in the secondary defense by presenting the viral antigen to circulating immune cells [36]. This is more likely to occur if there is exposure to a significantly high viral load, which increases the probability of transmission or infection; Langerhans cells, which act as antigen-presenting cells, are thought to facilitate HIV entry [40]. While present throughout the body's deeper skin layers, these cells are especially concentrated and closer to the surface on the mucosal lining of the foreskin and penile shaft. Male circumcision (MC) removes a significant number of these Langerhans cells. The remaining cells may be less prone to HIV uptake, likely because the skin surface becomes drier, hindering HIV particle attachment. This is supported by evidence suggesting that subpreputial moisture is a risk factor, as uncircumcised men with longer foreskins and consistently moist mucosal surfaces face a greater risk of HIV acquisition [41].

In sub-Saharan Africa, the growing acceptance of voluntary medical male circumcision (VMMC) has led to a slow increase in its prevalence, occurring alongside a slow decline in HIV prevalence attributed to various intervention strategies. Despite this, achieving sufficient VMMC coverage among men aged 15-49 years has been inconsistent, and reliable, up-to-date information on the current prevalence of male circumcision in these nations remains scarce [3].

Mills et al. conducted a meta-analysis of randomized controlled trials including 11,050 uncircumcised men in South Africa, Kenya, and Uganda to assess whether male circumcision prevents heterosexually acquired HIV infection. Using a random-effects model, they pooled relative risks, risk differences, and numbers needed to treat. Circumcision reduced incident HIV infection by 56%, with a pooled relative risk of 0.44 (95% CI: 0.33-0.60) and no important heterogeneity. The absolute risk reduction was 1.4% over about two years, corresponding to a number needed to treat of 72 men (95% CI: 50-143) to prevent one new HIV infection. The authors conclude that male circumcision is an effective prevention strategy, but its population impact depends on safe sexual behavior, counseling, cultural acceptability, avoiding risk compensation, and comprehensive integration with other HIV prevention interventions [33].

Weiss et al. conducted a systematic review and meta-analysis of observational studies assessing whether male circumcision reduces HIV-1 acquisition in men in sub-Saharan Africa. They identified 27 studies (cross-sectional, case-control, cohort, partner studies) from eight countries. Overall, circumcised men had about half the risk of HIV infection compared with uncircumcised men (crude pooled relative risk: 0.52; 95% CI: 0.40-0.68), which became stronger after adjustment for confounders (adjusted relative risk {RR}: 0.42; 95% CI: 0.34-0.54). Protection was greatest in high-risk groups such as sexually transmitted disease (STD) clinic attendees and truck drivers (adjusted RR: 0.29), but also present in general-population samples (adjusted RR: 0.56). The authors note heterogeneity and residual confounding as limitations, yet conclude that male circumcision likely has a substantial protective effect and merits evaluation as an HIV-prevention strategy in high-burden African settings [34].

Circumcision as a treatment for phimosis

Phimosis is defined as a condition in which the narrowed opening of the foreskin (or prepuce) makes it impossible to fully retract this skin layer from behind the glans penis [42]. Parents may bring children to the pediatrician with concerns about the non-retractability of the foreskin, often exhibiting high levels of anxiety. This situation often results in circumcision. Medical record analyses in England and Western Australia indicated that the rate of medically necessary circumcisions in children under 15 years was seven times higher than the expected incidence of true phimosis [43,44]. Physiologic phimosis involves a healthy foreskin tip that bulges when slightly pulled, with the narrowing located behind the tip. This is distinct from pathological phimosis, where slight pulling results in a cone shape with a white, scarred, and constricted foreskin tip and a very small meatal opening [45].

Approximately 96% of newborn males have a non-retractile foreskin, which is considered physiological phimosis. This is caused by natural adhesions between the foreskin and the glans, along with a narrow foreskin opening and a short frenulum ("frenulum breve"). The foreskin's retractability improves gradually over time, which can take anywhere from birth up to 18 years or more, and this process is facilitated by erections and the keratinization of the inner epithelial layer [46]. Preputial retractability increases as males age. However, even in otherwise normal males, 2% will never achieve full retractability [47-51].

Physiologic cases usually resolve with hygiene and gentle retraction, while topical steroid creams are effective, cheap first-line therapy for many pathologic cases. Circumcision completely removes the phimotic foreskin, cures phimosis, prevents recurrence, and reduces balanoposthitis and urinary tract infections [52].

Circumcision as a preventive measure

Urinary Tract Infection

Although much of the research on the link between circumcision and urinary tract infections (UTIs) is now at least ten years old, data still suggest that uncircumcised males have a higher incidence of UTIs during their first year of life [53]. This might be due to the presence of bacteria on the foreskin during the first six months of life, which could subsequently affect how easily the foreskin can be retracted [54].

Circumcision is a significant consideration for boys with vesicoureteric reflux (VUR). Given that UTIs frequently cause renal injury and scarring in VUR patients, preventing UTIs is a primary goal of treatment. A systematic review by Singh-Grewal et al., which analyzed 12 randomized and observational studies, found that circumcision significantly lowers the risk of UTIs. The pooled odds ratio was approximately 0.13 compared to uncircumcised boys. However, for otherwise healthy boys with a low baseline UTI risk (around 1%), the number needed to treat (NNT) to prevent a single UTI is high (about 111), suggesting limited net benefit for routine neonatal circumcision. Conversely, circumcision is more justifiable in high-risk groups, such as boys with recurrent UTIs or high-grade VUR, whose recurrence risks range from 10-30%. This translates to much lower NNTs (11 and 4, respectively). Since the complication rate is about 2% (primarily bleeding and infection), circumcision should be reserved for these high-risk boys rather than performed routinely [55]. A systematic review from 2013 estimated that the number needed to treat (NNT) to prevent a single urinary tract infection (UTI) in children with normal urinary systems was 4.3. The lifetime risk of a UTI was found to be 32% for uncircumcised males compared to 9% for circumcised males [56].

Penile Cancers

Epidemiological evidence suggests that male circumcision provides protection against penile cancer, given that this malignancy affects approximately 0.1% or less of men who are not circumcised [57]. Specifically, invasive penile cancer is strongly associated with phimosis, a condition where the foreskin cannot be fully retracted, which is naturally eliminated by the procedure of circumcision [58-60].

Studies indicate a connection between pediatric circumcision and a lower incidence of invasive penile cancer. Nonetheless, the evidence for a comparable link with in situ penile cancer or penile intraepithelial neoplasia is less conclusive [61]. A 2015 comparative analysis indicated that the frequency of penile cancer is comparable in the United States and Australia, despite the United States having significantly higher circumcision rates [62].

## Conclusions

Male circumcision remains a widely performed procedure driven by a combination of religious, cultural, and medical factors. From a surgical perspective, a variety of techniques - ranging from open methods such as the dorsal slit and forceps-guided resection to device-based approaches such as the Gomco clamp (Buffalo, NY: Goldstein Manufacturing Company), Plastibell, and other approved circumcision devices where available - allow providers to tailor the intervention to the patient's age and anatomy. Therapeutically, circumcision serves as the definitive treatment for pathologic phimosis, particularly when conservative measures fail, and offers a permanent solution for recurrent balanitis and paraphimosis. When performed by trained professionals under sterile conditions, it is a safe intervention with a low rate of serious adverse events.

Beyond its therapeutic role, robust evidence supports the use of circumcision as a vital preventive health measure. Most notably, it significantly reduces the risk of heterosexually acquired HIV by removing foreskin tissue rich in HIV-target cells, prompting global health organizations to recommend it as a key component of HIV prevention strategies. Additionally, the procedure provides demonstrated protection against other health risks, including invasive penile cancer and urinary tract infections, the latter being particularly beneficial for high-risk pediatric populations with urological abnormalities. Ultimately, continued research and proper implementation are essential to maximize these public health benefits while ensuring patient safety.
