# Correction: Male Circumcision Revisited: A Narrative Review of Techniques, Therapeutic Indications, and Preventive Benefits

**DOI:** 10.7759/cureus.c398

**Published:** 2026-01-30

**Authors:** Muhammad Rakib Hasan, Asmita Hossain, Nazmus Sakib

**Affiliations:** 1 Urology, Watford General Hospital, West Hertfordshire Hospitals NHS Foundation Trust, Watford, GBR; 2 Urology, Surrey and Sussex Healthcare NHS Trust, Redhill, GBR; 3 Gastroenterology and Hepatology, Square Hospitals Limited, Dhaka, BGD

This article has been corrected to align descriptions of PrePex and forceps-guided circumcision with current World Health Organization (WHO) guidance. Updates include revised safety language and replacement of references #22 and #24 to reflect updated WHO recommendations for elastic-collar compression devices, clarification of WHO-endorsed standards for forceps-guided circumcision, and corresponding changes to the Conclusions paragraph and Figure 1 callout text.

